# A Unique Case of Relapsed B-Acute Lymphoblastic Leukemia/Lymphoma as an Isolated Omental Mass

**DOI:** 10.1155/2014/425163

**Published:** 2014-10-16

**Authors:** Kanchan Kantekure, Furha Cossor, Kenneth B. Miller, Monika E. Pilichowska

**Affiliations:** ^1^Department of Pathology and Laboratory Medicine, Tufts Medicine Center, Boston, MA 02111, USA; ^2^Department of Hematology Oncology, Lahey Hospital and Medical Center, Burlington, MA 01805, USA; ^3^Department of Hematology Oncology, Tufts Medicine Center, Boston, MA 02111, USA

## Abstract

B-acute lymphoblastic leukemia/lymphoma (B-ALL) is a neoplasm of precursor cells committed to the B-cell lineage. Extramedullary involvement is frequent, with particular predilection for the central nervous system, lymph nodes, spleen, liver, and testis. We report an unusual case of B-ALL relapsing as an isolated omental mass along with bone marrow involvement.

## 1. Case

A 25-year-old man with history of B-ALL presented with subacute onset of abdominal pain and bloating in 06/2012.

He was initially diagnosed with B-ALL in 10/2009 based on peripheral blood and bone marrow biopsy findings, after presenting with fatigue, hepatosplenomegaly, and pancytopenia. Flow cytometry performed on peripheral blood showed a population of blast forms with weak expression of CD45 and coexpression of CD34, CD20, HLA-DR, CD19, CD10, and TdT. Cytogenetic analysis performed on peripheral blood and bone marrow aspirate by conventional karyotyping revealed normal male chromosome complement (46, XY) and was negative for BCR/ABL translocation. FISH was performed using cocktail of probes for the t (9; 22) (BCR-ABL1), t (8; 14) (IGH-MYC, CEP8), and 11q23 (MLL). He received induction chemotherapy with Cytoxan, vincristine, and prednisone followed by hyper-CVAD for consolidation. The follow-up interval bone marrow biopsies and peripheral blood smears on 11/2009 and 11/2010 did not show any evidence of leukemia. He received intensification therapy with methotrexate and L-asparaginase, followed by 9 months of 6-mercaptopurine, vincristine, methotrexate, and prednisone.

**Figure 1:**
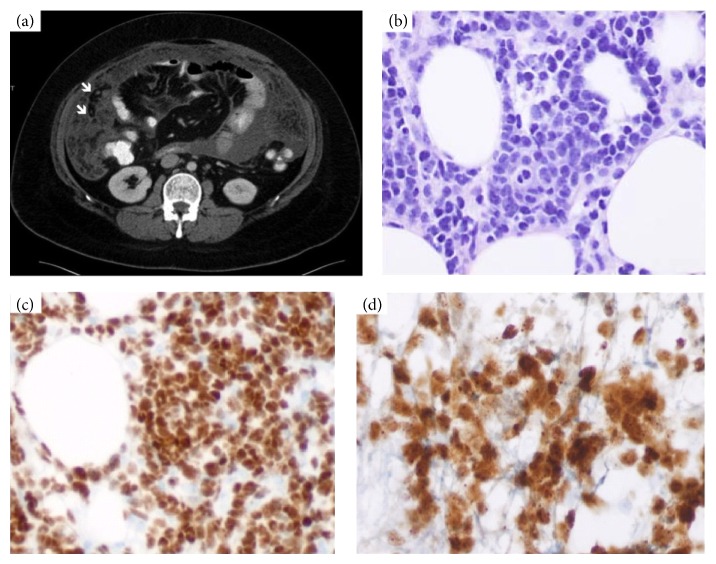
(a) CT scan of abdomen with contrast showed thickening of omentum and increased attenuation of the mesenteric fat (arrows) which overall is concerning peritoneal lymphomatosis. (b) H&E of omental biopsy with infiltration by small blast forms (×20). (c) Immunohistochemical stain for PAX-5 showed nuclear positivity in blast forms (×20). (d) Immunohistochemical stain for TdT showed nuclear positivity in blast forms (×40).

At presentation, CT scan of abdomen on 6/2012 showed heterogeneously high attenuation with greater omental infiltration as well as associated increased attenuation of the mesenteric fat, which overall was concerning peritoneal lymphomatosis and malignant ascites (Figure 1(a)). No other intra-abdominal organs were involved. CT-guided biopsy of the omentum revealed diffusely infiltrating blast forms (Figure 1(b)), which were positive for PAX5 (Figure 1(c)), TdT (Figure 1(d)), CD10, and CD20 by immunohistochemistry confirming diagnosis of relapsed B-ALL. Peripheral blood smear during relapse showed several atypical lymphocytes with large nuclei and scant cytoplasm did not have distinct nucleoli but were distinct in appearance from majority of small lymphocytes on smear. Subsequent bone marrow biopsy revealed involvement by B-ALL with 50% blasts and FISH detected trisomy 8. He was treated with cyclophosphamide, vincristine, adriamycin, and dexamethasone (hyper-CVAD). Due to quick regrowth between cycles, rituximab, bortezomib, and decitabine were added between cycles.

Recently in 2013, four years after initial diagnosis, patient presented with second bone marrow relapse and expired.

## 2. Discussion

The overall frequency of relapse in ALL is approximately 25% in children and 50% in adults, with a rate that is highly dependent on the immunophenotypic and genetic subtype or otherwise defined risk category of ALL [1–3]. Most ALLs relapse in the first 3 to 5 years from diagnosis. Only a very small percentage relapses more than 5 years from diagnosis, and relapses may occur 10 to 20 years later in a minority of patients. Extramedullary involvement by B-ALL is frequent. Relapsed ALL may involve the bone marrow or extramedullary tissues, with particular predilection for the central nervous system, lymph nodes, spleen, liver, ovary, and testis [4–8].

Isolated omental presentation or relapse of B-ALL is not described in the literature. Subpopulations of blasts able to migrate to these areas may have a survival advantage and give rise to disease recurrence [8]. Early reports on the dissemination of tumor cells in peritoneal tissues after intraperitoneal inoculation document the infiltration of sites rich in “milky spots,” such as the omentum, mesentery, and gonadal fat [9, 10]. Omental stroma shows the constitutive expression of IL-7, FLt3 ligand, and CXCL12, which are potent cofactors for the growth of B cell precursors [11].

Literature speculated that tissues like the omentum and ovary attract the B-ALL cells with higher 5T4 oncofetal antigen transcript. Bone marrow from relapse patients has a significantly higher percentage of 5T4 positive leukemic blasts [8]. This interaction occurs through the 5T4/CXCR4 dependent chemotaxis axis and/or with enhanced adhesion of the elevated levels of VLA-4 (very late antigen-4) on these leukemic cells to VCAM-1 (vascular cell adhesion molecule 1) expressed by the microenvironment [12–14].

## References

[1] Pui C. H., Pui C. H. (2006). Acute lymphoblastic leukemia. *Childhood Leukemias*.

[2] Faderl S., Jeha S., Kantarjian H. M. (2003). The biology and therapy of adult acute lymphoblastic leukemia. *Cancer*.

[3] Kantarjian H., Thomas D., O’Brien S., Cortes J., Giles F., Jeha S., Bueso-Ramos C. E., Pierce S., Shan J., Koller C., Beran M., Keating M., Freireich E. J. (2004). Long-term follow-up results of hyperfractionated cyclophosphamide, vincristine, doxorubicin, and dexamethasone (Hyper-CVAD), a dose-intensive regimen, in adult acute lymphocytic leukemia. *Cancer*.

[4] Moorman A. V., Ensor H. M., Richards S. M., Chilton L., Schwab C., Kinsey S. E., Vora A., Mitchell C. D., Harrison C. J. (2010). Prognostic effect of chromosomal abnormalities in childhood B-cell precursor acute lymphoblastic leukaemia: results from the UK Medical Research Council ALL97/99 randomised trial. *The Lancet Oncology*.

[5] Jacobs J. E., Hastings C. (2010). Isolated extramedullary relapse in childhood acute lymphocytic leukemia. *Current Hematologic Malignancy Reports*.

[6] Krishnan S., Wade R., Moorman A. V., Mitchell C., Kinsey S. E., Eden T. O. B., Parker C., Vora A., Richards S., Saha V. (2010). Temporal changes in the incidence and pattern of central nervous system relapses in children with acute lymphoblastic leukaemia treated on four consecutive Medical Research Council trials, 1985–2001. *Leukemia*.

[7] Reid H., Marsden H. B. (1980). Gonadal infiltration in children with leukemia and lymphoma. *Journal of Clinical Pathology*.

[8] Lo Nigro L., Cazzaniga G., Di Cataldo A., Pannunzio A., D'Aniello E., Masera G., Schiliró G., Biondi A. (1999). Clonal stability in children with acute lymphoblastic leukemia (ALL) who relapsed five or more years after diagnosis. *Leukemia*.

[9] Hagiwara A., Takahashi T., Sawai K., Taniguchi H., Shimotsuma M., Okano S., Sakakura C., Tsujimoto H., Osaki K., Sasaki S., Shirasu M. (1993). Milky spots as the implantation site for malignant cells in peritoneal dissemination in mice. *Cancer Research*.

[10] Tsujimoto H., Takahashi T., Hagiwara A., Shimotsuma M., Sakakura C., Osaki K., Sasaki S., Shirasu M., Sakakibara T., Ohyama T., Sakuyama A., Ohgaki M., Imanishi T., Yamasaki J. (1995). Site-specific implantation in the milky spots of malignant cells in peritoneal dissemination: immunohistochemical observation in mice inoculated intraperitoneally with bromodeoxyuridine-labelled cells. *The British Journal of Cancer*.

[11] Pinho M. D. F. B., Hurtado S. P., El-Cheikh M. C., Borojevic R. (2005). Haemopoietic progenitors in the adult mouse omentum: permanent production of B lymphocytes and monocytes. *Cell and Tissue Research*.

[12] Castro F. V., McGinn O. J., Krishnan S., Marinov G., Li J., Rutkowski A. J., Elkord E., Burt D. J., Holland M., Vaghjiani R., Gallego A., Saha V., Stern P. L. (2012). 5T4 oncofetal antigen is expressed in high risk of relapse childhood pre-B acute lymphoblastic leukemia and is associated with a more invasive and chemotactic phenotype. *Leukemia*.

[13] Imai Y., Shimaoka M., Kurokawa M. (2010). Essential roles of VLA-4 in the hematopoietic system. *International Journal of Hematology*.

[14] Rettig M. P., Ansstas G., Dipersio J. F. (2012). Mobilization of hematopoietic stem and progenitor cells using inhibitors of CXCR4 and VLA-4. *Leukemia*.

